# In the name of the family? Against parents’ refusal to disclose prognostic information to children

**DOI:** 10.1007/s11019-021-10017-4

**Published:** 2021-04-13

**Authors:** Michael Rost, Emilian Mihailov

**Affiliations:** 1 Institute for Biomedical Ethics, University of Basel, Bernoullistr. 28, 4056 Basel, Switzerland; 2 Research Centre in Applied Ethics, Faculty of Philosophy, University of Bucharest, Bucharest, Romania

**Keywords:** Pediatric oncology, Family unit, Prognostic disclosure, Systemic thinking, Decision-making

## Abstract

Parents frequently attempt to shield their children from distressing prognostic information. Pediatric oncology providers sometimes follow parental request for non-disclosure of prognostic information to children, invoking what we call the stability of the family argument. They believe that if they inform the child about terminal prognosis despite parental wishes, cohesion and family structure will be severely hampered. In this paper, we argue against parental request for non-disclosure. Firstly, we present the stability of the family argument in more detail. We, then, set out the (conceptual, legal, systemic) entitativity of the family and the kind of value the stability of the family argument assumes, before we set on to critically evaluate the argument. Our analysis shows that disclosure of prognostic information to children does not necessarily destabilize the family to a greater extent than non-disclosure. In fact, a systemic perspective suggests that mediated disclosure is more likely to result in a (long-term) stability of the family than non-disclosure. It is in the interest of the family to resist the initial aversive reaction to delivering bad news. In the final part, we draw a set of recommendations on how to facilitate decision-making in face of parental request for non-disclosure.

## Introduction

When a child is diagnosed with cancer, every family member will face the situation. The diagnosis may freeze them to openly discuss the prospects and how everyone is feeling. Still, for clinical communication, international guidelines recommend sharing treatment-related decision-making (DM) among children, parents, and health professionals (Levetown 2008; American Medical Association 2019; Katz and Webb 2016), which includes disclosure of the prognosis. Children are usually deemed incapable of fully autonomous judgment, so parents act as surrogates (Katz and Webb 2016). However, parental authority can be limited by a child’s best interests, vulnerability (Gheaus 2018), and child welfare legislation (Cummings and Mercurio 2010).

Withholding information from children not only means that they are excluded from respective DM, but also that they are deprived of the opportunity to actively develop their decisional capacity. We found in our research that information about the prognosis of cancer was sometimes withheld from children due to explicit parental wishes (Rost et al. 2018a, 2019). Respective children were 7, 9, 10, 11, and 16 years of age (Rost et al. 2018a). It is not a surprise that parents often attempt to shield their children from what they think is emotionally overwhelming. What attracted our attention was the reasoning behind pediatric oncology providers’ decision to follow the parental wish. They respected the parental wishes on the contention that “open disclosure would make interactions more difficult” (Sisk et al. 2016, p. 3). They were “concerned about the effect this revelation may have on relationships with parents, brothers and sisters, other relatives” (Agranoff and Mauer 1965, p. 231). Their reasoning is that if they inform the child about terminal prognosis despite parental wishes, cohesion and family structure will be severely hampered.

In their estimation, the risk of destabilizing the family unit outweighed the benefits of disclosing the prognosis to the child. Consequently, children were not informed about the prognosis. We will call this rationale the “stability of the family argument” because it favors the inner functioning of a family. In this respect, our empirical findings point towards a particularity of pediatric DM that health professionals sometimes find it harder to go against parents’ wishes.

The image of the family as a unified body is regularly applied to prognostic disclosure in the pediatric setting. Various characteristics of the family as a whole are highlighted as determining considerations for shaping DM: “autonomy of the family unit” and “overall family goals” (Katz and Webb 2016), “importance of the family unit” (Cummings and Mercurio 2010), “family situation” and “sovereignty of the family unit” (Jeremic et al. 2016; Weiner et al. 2020), “psychosocial recovery of the family” (Whittam 1993), “family privacy” (Lantos 1996), “stable system of social support provided by the family” (Sigman et al. 1993), “family structure” and “family considerations” (Sisk et al. 2016), and “effects of a decision on all family members” (Harrison et al. 1997). Historically, the concern that “disclosure could upset the family structure” has been apostrophized as one argument against disclosure (Sisk et al. 2016). Furthermore, another own study on the same sample of children with cancer revealed that the burden of cancer treatment had a great impact on an entire family’s daily life (Rost et al. 2018b).

Family considerations play a ubiquitous role in pediatric moral thinking. But does pediatric oncology providers’ family argument *eo ipso* justify withholding prognostic information from children? In this paper, we critically evaluate the stability of the family argument. We do not deal with the question of whether the child should have the final say. Also, we do not focus on mature minors, who, in Switzerland for example, possess the legal and ethical authority to consent to medical treatment irrespective of their age (Swiss Civil Code, Art.16) (Schweizerische Akademie der Medizinischen Wissenschaften 2017; Peter 2008), and who are sometimes exempt from the requirement of parental consent in other countries, too (Coleman and Rosoff 2013). Finally, it has to be stressed that a child’s age and development determine the assessment of disclosure. A quantitative study on children’s involvement in decision-making has shown that pediatric oncology providers deemed the majority of children older than 9 years of age capable of understanding prognosis and the majority of children older than 11.5 years of age capable of making treatment decisions (Rost et al. 2017a). As such, disclosing prognostic information to a 6 years old child encompasses different factors than to a 17 years old child. Still, our evaluation of the stability of the family argument applies to cases in which children are capable of understanding some information related to their illness (e.g. diagnosis, response to treatment, prognosis).

At the heart of our analysis is the stability of the family argument as it is illustrated by pediatric oncology providers in our empirical study (and in other studies) when they justify withholding information about prognosis from a child (Rost et al. 2018a; Sisk et al. 2016; Agranoff and Mauer 1965). While parental requests for non-disclosure might be more likely in cases of a terminal prognosis, our analysis applies to the entire spectrum of childhood cancer prognoses. Furthermore, we concentrate on prognostic information disclosure against the backdrop of a (presumed) destabilization of the family unit. This is not to say that pediatric oncology providers solely consider the possible destabilization of the family unit when confronted with parental non-disclosure requests. In fact, there are many patient and family considerations (to be) taken into account, such as patients’ communication styles and individual needs, a family’s cultural background and illness experience, or the child-parent relationship (Sisk et al. 2016). Finally, we acknowledge that prognostic disclosure can take various forms along a continuum ranging from no disclosure at all to full disclosure.

After presenting the stability of the family argument in more detail, we will set out the (conceptual, legal, systemic) entitativity of the family and the kind of value it assumes. We will, then, evaluate the family argument, before, in the final part, we will draw a set of recommendations on how to facilitate maneuvering through the difficulties of parental non-disclosure requests. This is crucial because pediatric oncology providers find it important to increase their moral competencies in challenging situations, such as poor prognoses and family’s feelings of shock (Weiner et al. 2020).

## The stability of the family argument

While pediatric oncology providers do not spell out the stability of the family argument in a formal-logical way, it can be reconstructed as follows. Their rationale to follow the parental wish not to disclose prognostic information to their children is based on one normative and two descriptive premises:

- *P1*_descr._*—A family of a child with cancer is particularly in need of stability.*
- *P2*_norm._*—Pediatric oncology providers should contribute to the preservation of the inner stability of the family.*
- *P3*_descr._*—Disclosing prognostic information to the child against the parental wish will further destabilize the family unit.*
- *C—Pediatric oncology providers should not disclose prognostic information against the parental will.*

The premises have assumptions about vulnerability and allocation of responsibilities. Firstly, pediatric oncology providers assume that a family of a child with cancer is particularly in need of stability in order to maneuver through such difficult times. The inner stability of the family unit is considered fragile and composed of numerous domains, such as social, emotional, cultural, physical, or economic ones. Secondly, pediatric oncology providers see themselves entitled, if not responsible, to contribute towards the preservation of the inner stability of the family amid a life-threatening disease of a child. In their view, they should facilitate the inner stability of the family whenever possible, because this is part of their holistic care for a child. They believe that disclosing the prognostic information to the child, despite parental wishes not to do so, is going to further destabilize the family and, ultimately, puts their inner functioning at risk.

The anticipated destabilization of the family as a consequence of information disclosure has been described in the literature for both the adult and the pediatric setting. Usually, family members attempt to protect the patient from harm. They do so by avoiding the burden to openly discuss the prognosis and to have to cope with the patient who was harmed by the prognosis. For the adult setting, a patient’s emotional distress, incapacity to cope with the information, a deteriorating physical condition due to an emotional burden, hopelessness, despair, and psychological morbidity, have been identified as justifications for non-disclosure of prognostic information (Mitchison et al. 2012; Lee and Wu 2002; Shahidi 2010). For the pediatric setting, naturally, parents of children with cancer want to live up to their role as protectors and caregivers (Matsuoka and Narama 2012; Bluebond-Langner et al. 2010). They, thus, exhibit an even stronger motive to protect and care for their child by withholding prognostic information as compared to family members of adult cancer patients. When they withhold prognostic information, parents give the following reasons: discussing the disease burdens the child, making the child’s life easier, causing anxiety and distress, protecting the child from the life-threatening nature of the condition and placing undue mental or physical strain on the child (Whittam 1993; Badarau et al. 2015; Clarke et al. 2005; Claflin and Barbarin 1991; Fukuda and Fukuda 2018; Coyne et al. 2016).

The stability of the family argument implies certain assumptions about the entitativity and the values of the family. Firstly, the argument is predicated on the (ontic) nature of the family as a whole, that is an entity. This is echoed by clinical ethics guidelines on DM in the pediatric setting: “a family-centered approach considers the effects of a decision on all family members, their responsibilities toward one another and the burdens and benefits of a decision for each member, while acknowledging the special vulnerability of the child patient.” (Harrison et al. 1997, p. 826). In this vein, pediatric prognostic disclosure affects the entire family and implies the recognition of the entitativity of the family. Secondly, the argument appropriates the view that a family and its inner functioning needs to be protected. While children’s wellbeing is of value and each family member has rights, the question is whether the value of family is more than the mere aggregate of individual values. Both the entitativity and values of the family will be addressed in the next section.

## Entitativity and value(s) of the family

The wholeness of the family is frequently emphasized in pediatric DM. Accordingly, a family represents a unit that transcends its members and is regularly perceived as an entitative group. We will now present different types of entitativity of the family, namely the family as a legal, moral, and systemic entity. By explaining the specificity of each type of family entitativity, we will clarify the conceptual resources of the family argument. Finally, we will carve out the kind of value assumed in the family argument.

### Legal entitativity

According to international law, a family is an entity with legal rights. The Universal Declaration of Human Rights stipulates that “no one shall be subjected to arbitrary interference with his family” and that “everyone has the right to the protection of the law against such interference” (Art. 12) (United Nations 1948); we find the same in the United Nations’ International Covenant on Civil and Political Rights (Art. 17) (United Nations 1966). Furthermore, the United Nations’ International Covenant on Economic, Social and Cultural Rights demands that “the widest possible protection and assistance should be accorded to the family” (Art.10) (United Nations 1976). At a European level, the European Convention on Human Rights grants the right to found a family (Art. 12) (European Court of Human Rights-Council of Europe 2010). Thus, at the level of international law, the family represents a legal entity.

### Moral entitativity

Apart from their status as a legal entity, families are often treated as an entity with moral status. Cutas and Smajdor demonstrate how many public policies treat the family as an entity towards you can have moral obligations (Cutas and Smajdor 2017). In the contexts of human rights legislation, fertility treatments, public health, sociology and social work, the family is treated as an entity. It has needs and interests that are not reducible to the needs and interest of its members. More importantly, however, the authors expose that a family’s and its individual members’ interests can conflict with each other and that the moral status of the family can supersede that of its members.

It seems as if there is a (legislative and institutional) commitment to the integrity and continuation of the family, sometimes even at the expense of individual family members’ safety and well-being. What the moral entitativity view recognizes is not an alignment of interests within the family unit, which can be achieved through consensus, but a supra-individual interest in the continued existence of the family as an entity. Cutas and Smajdor rightly point out that a moral status of the family which supersedes individual members’ rights can hardly be reconciled with contemporary western bioethics’ emphasis on individuals’ best interests (Cutas and Smajdor 2017).

### Systemic entitativity

Systemic therapy represents a major therapeutic direction that is characterized by a plethora of theoretical underpinnings and therapeutic approaches (Helle 2019). It originated from the interdisciplinary field of systems theory which assumes that “the behavior of a system can only be understood by considering the individual characteristics of elements within the system, and the relationship between these elements (Tickle et al. 2015, p. 124).

In systemic therapy, an individual’s psychological disorder is not primarily a pathology of the respective person which can only be treated and cured within that person, but a reaction to a dysfunctional social system—most frequently the family (Helle 2019). Accordingly, systemic therapy emphasizes the social context of psychological disorders. The patient is seen as the “symptom carrier”—the social system (e.g. family) has a psychological disorder, not the patient (Kowalsky 2012). Therefore, the therapeutic process aims to enhance the ability of the social system to function adaptively and to allow growth by developing new patterns of communication, interaction, relationships, and behavior (Kowalsky 2012). Hence, it is the family (i.e. a system of people) that is addressed by systemic therapy and which is understood as a systemic entity whose inner functioning and (in)stability is one focal point of attention. Resolving problems of a person, from this perspective, means to start thinking from the family as a systemic entity which co-determines a person’s psychological condition.

### The family as a systemic entitativity

The previous three examples illustrate what kind of entity the family can be, namely a legal entity with legal rights, a moral entity with interests, and a systemic entity with inner functioning and stability. It is primarily the systemic entitativity that is crucial for our critical examination of the stability of the family argument. The legal entitativity is not directly relevant for prognostic disclosure in pediatric oncology, but hints at how the larger normative background influences pediatric practice. Being legally accountable might inhibit physicians to get involved in complicated cases and increase the likelihood to follow parental wishes. Moral entitativity applies to some extent. While there is no discernable interest of the family as a unit—rather potential disunity of interests, there is an interest to preserve the family as a unit that requires moral consideration. But this interest is supra-individual and it is more concerned with the continued existence of the family as an entity or with bringing into existence such entities (e.g. through fertility policies), rather than with the inner dynamics.

The systemic entitativity directs us to the crux of the matter: the interdependence of familial relationships understood as a (social) system and what inner functioning is about. Thus, the family is “an actually lived set of family bonds, which are characterized through the diverse experiences of attachment between the individual family members.” (German Ethics Council 2014, p. 37). Physicians’ reference to a family’s inner functioning targets the lived familial relationships of a child and their inner decisional dynamics, how the family members interpret and react to the harsh facts of a cancer diagnosis. Hence, understanding the family as a lived set of family relationships applies to our examination of the stability of the family argument.

### Value(s) of the family

The family is widely considered “the natural and fundamental group unit of society” (Universal Declaration of Human Rights, Art. 16) (United Nations 1948). But what value of the family pediatric oncology providers attempt to protect when they invoke the stability of the family argument? We can distinguish between two types of values.

Firstly, the family is often construed as a social institution that has to be protected to maintain the social value of the family (German Ethics Council 2014). In this regard, taking care and protecting the family are instrumentally valuable for social harmony and prosperity. Stable families contribute to stable societies. The social value of family is based on an indirect pattern of collective benefits by promoting and protecting private family ends. Society is best served when we encourage individuals to dedicate their resources to their closest relatives.

Secondly, the relationships among the family members themselves have to be protected (German Ethics Council 2014). Trusting bonds and collective flourishing within the family may have social benefits, but they are also valuable in themselves. Here, the inner functioning and the genuine roles of the family members have intrinsic value and, hence, have to be shielded from the considerable strain. The intrinsic value of family relationships closely resembles what pediatric oncology providers refer to when they vindicate non-disclosure of prognostic information by (presumed) stabilization of the family unit. That is, pediatric oncology providers primarily consider the stability of the family an intrinsic value. They do not refer to it as a value that is instrumental in supporting other factors. Such a view is in line with legal documents which grant families the right to be protected from undue interference. In what follows, we will analyze whether parental authority should be respected based on the family argument.

## Examining the family argument

We have pointed out the family argument’s (a) underlying ontological view (systemic entity), and (b) normative point of reference (intrinsic value of family relationships). We will now turn to the question of whether withholding prognostic information from children because of explicit parental wishes is justified by the stability of the family argument. We will analyze whether the three premises are supported by relevant normative guidelines and empirical evidence.

### P1_descr._: A family of a child with cancer is particularly in need of stability

A child’s cancer disease and related DM is fraught with psychological distress for the entire family (i.e. children, parents, siblings) (Theunissen et al. 2007; Rosenberg et al. 2013; Klassen et al. 2007; Grootenhuis and Last 1997; Robinson et al. 2006; Phipps et al. 2005a; Packman et al. 2010). Moreover, a child’s cancer diagnosis marks a loss of a family’s normal life as it limits opportunities to engage in leisure activities, interrupts existing friendships, affects working lives, and causes financial burden (Rost et al. 2018b; Badarau et al. 2015; De Clercq et al. 2016; Bjork et al. 2005; Di Battista et al. 2017; Griffiths et al. 2011; Warner et al. 2015). A family’s ability to cope with the cancer-related burden highly depends on its inner stability. The higher the burden, the more inner stability is required for functional coping strategies. This is because coping requires open communication and adaptive change which, in turn, are more likely to materialize the more stable the family unit is. For example, family cohesion and family functioning are protective factors that advance children’s and parents’ health (Huang et al. 2018; Alderfer et al. 2009; Pelcovitz et al. 1998; Phipps et al. 2005b). Furthermore, maintaining and re-establishing the stability of the family reduces family distress and enhances individual members’ quality of life (Kobayashi et al. 2014; Kelly and Ganong 2011; Long et al. 2014). In this sense, a family’s stability is a prerequisite for its individual members’ and its overall coping and well-being. Thus, the claim that a family of a child with cancer is particularly in need of stability is true. Note that premise 1 rests on systemic entitativity, since it assumes the wholeness and inner functioning of a family.

### P2_norm._: Pediatric oncology providers should contribute to the preservation of the inner stability of the family

For children with life-limiting and life-threatening illnesses (e.g. cancer) international guidelines universally recommend that palliative care ought to begin at diagnosis (World Health Organization 1998; American Academy of Pediatrics 2013; Craig et al. 2007; Association for Children’s Palliative Care 2009; Canadian Hospice Palliative Care Association 2006; EAPC Taskforce for Palliative Care in Children 2009). The same guidelines unanimously require pediatric oncology providers to collaborate not only with the child but also with the family. Besides, it is recommended to provide physical, psychological, social, and spiritual care for children and their families (Rost et al. 2017b). This kind of holistic care certainly embraces being attentive to a family unit’s (de)stabilization. The psychosocial healthcare needs of children and their families should be systematically assessed as a standard of care in pediatric oncology (Kazak et al. 2015). Hence, pediatric oncology providers correctly perceive their role as co-facilitator of the inner stability of the family. That being said, the stability of the family argument does not primarily address the family as an institution that is a fundamental pillar of society, but rather the value of protecting the autonomy of family relationships against external interventions. As the American Academy of Pediatrics states, “in pediatric care we often need to expand our understanding of autonomy to recognize the autonomy of the family unit, allowing respect for both the privacy of the family unit, within limits, and parental authority and responsibility for medical decision-making” (Katz and Webb 2016, p. 2). Premise 2 is, thus, in line with pediatric care guidelines. Finally, premise 2 acknowledges the inner functioning of the family as an intrinsic value that needs to be protected.

### P3_descr._: Disclosing prognostic information to the child against parental wish will further destabilize the family unit

The inner functioning of a family in face of a child’s cancer prognosis is determined by numerous factors that go beyond the mere stability of the family unit, such as a family’s value system and illness understanding, external coping resources, family members’ resilience or communication styles within the family. This premise, however, targets the potentially adverse impact of disclosing prognostic information against parental will on the family unit. We challenge pediatric oncology providers’ conviction about the destabilizing consequences of disclosure on numerous grounds (Cole and Kodish 2013).

Firstly, children are very perceptive of their medical reality. They are not clueless as the adults may want them to be. The child is a spectator “of the hushed whispers and discussions among grown-ups and can ascertain that a secret exists that is not to be discussed” (Sisk et al. 2016; Claflin and Barbarin 1991; Coyne et al. 2016; Cole and Kodish 2013, p. 641; Bluebond-Langer 1980; Lin et al. 2019). Children know when something is wrong (Wangmo et al. 2016). If they already perceive their medical uncertain condition, then disclosing prognostic information may clarify the reality of “hushed whispers”.

Secondly, it is more frightening to be left wondering because “fantasies are often worse than realities” (Wangmo et al. 2016, p. 15). Not disclosing information may lead to adverse outcomes on the part of the child, who may construct partial or distorted explanations, which, ultimately, increases emotional distress (Kunin 1997). There is evidence that limited or non-disclosure did not shield children from emotional distress (Claflin and Barbarin 1991). Children may even imagine a different, sometimes worse scenario (Clarke et al. 2005; Vernick and Karon 1965). They trust their parents to share illness-related information (Coyne et al. 2016), which, in the case of withheld information, is breached. Lastly, disparate information can cause confusion and anxiety on the part of the child, and restricting information might even increase (not mitigate) uncertainties and anxiety (Coyne et al. 2016).

Thirdly, the child (and the family) benefits from disclosing information. For example, it provides the opportunity to develop their decisional capacity (Lin et al. 2019), it causes children to value more strongly their treatment (Fukuda and Fukuda 2018), it promotes the therapeutic relationship and compliance with treatment recommendations (Hudson et al. 2019; Slavin et al. 1982), it gives the child some control over the situation and a validation of the experience (Lin et al. 2019; Kunin 1997), it fosters trust in the physician (Lin et al. 2019; Kunin 1997; American Academy of Pediatrics 1995), it improves a child’s well-being (Bluebond-Langer 1980; Hudson et al. 2019), it generally increases the likelihood of children approaching their parents for information (LePoire 2006), and it empowers children by enhancing their assertive agency (Lin et al. 2019).

Fourthly, we also need to take into account how disclosure versus non-disclosure affects trusting relationships. Filtering related information and, thereby, monitoring the nature and extent of disclosed information can prevent an erosion of trust in some cases (Ruhe et al. 2016). Generally, it is recommended to shape the pediatric disclosure of a prognosis in a developmentally appropriate way (Levetown 2008; American Medical Association 2019; Katz and Webb 2016). While withholding information is an extreme form of filtering information, pediatric oncology providers could actively work towards a lower-threshold filter. Such a compromise between physicians and parents, in the form of filtered prognostic disclosure, might achieve both respecting parental authority and children’s right to know. Ultimately, trusting parent–child, child–physician, and parent–physician relationships could be maintained. This perspective also warns against an oversimplifying dichotomization of disclosure into “tell” versus “don’t tell” and underlines the gradual nature of prognostic disclosure.

In light of all this, we have reasons to believe that the provision of appropriate information is helpful for young patients (Coyne et al. 2016; Lin et al. 2019; Ishibashi 2001; American Academy of Pediatrics 2000). To put it cautiously, the current evidence does not favor the estimation that the child—as the focal family member—will be mainly negatively affected by disclosure and will not be positively affected by non-disclosure. On the contrary, it documents numerous ways in which disclosing information can be positively integrated.

Still, it can be argued that disrespecting parental wishes forces the family into a discussion that it does not want. And this leads to an erosion of trust between the family and the staff, which ultimately destabilizes the (anyway highly fragile) family unit. Indeed, parents’ trust in the provider–parent relationship can decrease when pediatric oncology providers fail to understand parental needs (Mack et al. 2017). While trust-building requires time and trust has to be earned by providers (Mack et al. 2017; Baenziger et al. 2020), once established it is crucial on many levels. Parents are more likely to follow providers’ recommendations (Mack et al. 2017; Baenziger et al. 2020; Sisk et al. 2020). Also, trust helps build relationships between providers and parents that provide “a relational context in which other interpersonal communication occurred” (e.g. exchanging information, making decisions) (Sisk et al. 2020, p. 1). Lastly, parental distress appears to be associated with lower quality of life of children (Bakula et al. 2020; Pierce et al. 2017), which underscores the importance of a trusting parent–provider relationship from the perspective of the child.

Yet, respecting parents’ refusal of prognosis disclosure is not neutral with regards to undermining trust. While not being intrusive may preserve some level of trust, the lack of engagement with the challenges of a situation could undermine a trusting environment (Mihailov and Savulescu 2018). There is a cost of omission which cannot be ignored simply by hoping that unqualified respect for parental wishes will lead to greater trust in the end. When taking into account the entire family, it becomes clear that in both cases—disclosure and non-disclosure—negative outcomes will materialize for individual family members and, thus, for the family unit. Given empirical evidence and experts’ opinion, neither unmediated disclosure nor complete non-disclosure appears to be the ideal path. Importantly for our analysis, disclosure of prognostic information to children does not necessarily destabilize the family to a greater extent than non-disclosure. Through careful intervention, it is more likely to stabilize a stressed family.

We need to look at the potential consequences of (non)disclosure through the lens of systemic thinking if we are to understand the family’s inner dynamics. On the one hand, systemic thinking highlights the importance of the family unit for a child’s health and well-being. This seems to suggest non-disclosure of prognostic information to spare the family the burden of discussing and integrating the prognosis. On the other hand, for systemic thinking open communication and openness to change are necessary conditions for a family to adapt and thrive, which eventually facilitates stability and cohesion. Familial relationships could become problematic, if members operate with fixed patterns of, for example, blame or manipulation (Finlay 2016). In such closed systems, the “change appearing in one person is met with a compensating action by another, and this has the effect of maintaining the status quo” (Finlay 2016, p. 214). But reactive and fixed patterns of response ultimately impede the holistic change of the family as a whole.

Imagine a situation where parents display negative attitudes towards prognostic disclosure at home, which are noticed by the 15-years-old brother of the patient. When talking to pediatric oncology providers in the hospital, this sibling is at risk to articulate a biased view of the patient’s capacity to engage in DM surrounding prognosis, thereby abetting the respect for the parental wish not to disclose a prognosis to the patient. Or the sibling internalizes the parental attitudes resulting in a hesitancy to discuss the cancer-experience with his parents (or other significant others) because of low illness-related self-efficacy. This scenario might result in non-disclosure and seems to fulfill the goal of shielding the patient and family members from emotional distress. In the long run, however, they consolidate dysfunctional familial patterns of communication, interaction, relationships, and behavior. More critically, they inhibit the adaptive change of the entire family. The patient is left wondering and does not know what is wrong. The sibling has difficulties in addressing his feelings and thoughts. And, consequently, the parents cannot respond to the siblings’ needs because they do not know them.

Systemic thinking reveals that what is needed to maintain the stability of the family unit is open communication about family dynamics and flexible familial roles. For example, parents will have to accommodate their role towards both the ill child, who needs more attention and time, and siblings, who may feel left alone with their worries; parents will have to be aware that their couple-relationship will endure immense pressure. The situation requires an adaptive change to maintain a family’s inner functioning. Refusing change to maintain a family’s cohesion is dysfunctional from a systemic therapy perspective. The rationale of preserving a family’s inner functioning could be a mask for preserving the *status quo* and prioritizing the parental preferences that refuse the aversive medical reality.

Not disclosing prognostic information to the child excludes the child (and, most likely, siblings), and, consequently, eliminates the opportunity to exercise the child's agency as part of wellbeing. The family system perspective warns that intrafamilial relationships will be dysfunctional if we do not know the child’s preferences and values in disclosure. Knowing the child’s preference is facilitated by the adaptive change of the entire family and it helps parents to decide what roles they can fulfill and when. The child’s preference for disclosure, non-disclosure, or partial disclosure informs the parents when to be protectors and when to be mediators who filter information.

From the family system perspective, we should avoid problematic intrafamilial relationships. For example, in imagining a necessity to hide negative medical information, parents can experience feelings of guilt, and the child can experience feelings of betrayal, hidden loyalties between co-conspiring siblings and parents can lead to self-incongruence, or siblings can suffer from parentification (i.e. role reversal that causes a child to function as a parent). In the short-term, this can have the envisaged benefit of maintaining the inner functioning of the family (i.e. the *status quo* before the revelation of a prognosis). However, this short-term benefit is only apparent because it renders less likely any adjustment and coping of the family as a whole. In the mid-term and long-term, it increases the likelihood of dysfunctional family patterns and resulting anxiety for family members, which is so much harder to deal with and overcome. Conversely, allowing the entire family to adjust and facilitating the respective process will strengthen the family unit and its coping ability. A strengthened family unit and coping ability are likely to persist beyond a child’s possible death and, thereby, also helps the family to thrive in the long run. This is why the option of non-disclosure is often based on an incomplete assessment of the situation, namely the first phase of prognosis disclosure in which family members react negatively to bad news.

Related to this, it has been evidenced that surrogate DM, in one-third of the cases, incorrectly predicts patients’ end-of-life preferences (Shalowitz et al. 2006). Such a margin of error points towards the inherent possibility of parental misjudgments of children’s preferences and best interests. Still, parental judgments are the basis for their explicit wish not to disclose prognostic information, which, in the end, elicited pediatric oncology providers’ family argument. That means, not only the conclusion of the family argument is false (because one of its premises is false), its origin might be incorrect in some instances. The parental judgments about a child’s best interest might be mistaken already.

Premise 3, thus, does not hold. Conveying prognostic information against parental wishes can have destabilizing consequences on the family unit when it is done without due care, but, even more, it can have the opposite impact. A systemic perspective suggests that mediated disclosure is more likely to result in a (long-term) stability of the family than non-disclosure. Conceptualizing the family as a systemic entity not only reveals the importance of open communication and openness to change but highlights the interdependent familial relationships which determine the stability of the family.

## Making progress with moral disagreement on family values

Our rejection of the stability of the family argument is not an attack on family values. Indeed, we argued against parents’ request for non-disclosure of prognostic information to children. Not taking parental refusal at face value looks similar to liberal approaches that favor the rights and best interests of the children against parental authority. By contrast, traditional views of the family see parental authority over children in ways that cannot be reduced to the best interests of their children, and it can limit children’s autonomous choices (Engelhardt 2010).

However, we did not dispute the normative premise of the family argument, nor did we denied the empirical premise that a family of a child with cancer is in need of stability. We did not criticize traditional family values from a liberal perspective. On the contrary, we grant that family relationships of trust and collective flourishing are intrinsically valuable. Moreover, we show that this is in line to some extent with pediatric practice and guidelines. What we did was to empirically scrutinize the pivotal premise that disclosing prognostic information to the child against the parental wish will further destabilize the family unit. By turning the argument on its head, we show that stability considerations themselves warrant us to involve children in pediatric prognosis disclosure and resist parental refusal in the first instance.

This means, more generally, that resisting parental request for non-disclosure does not necessarily reflect a deep conflict between liberal values and traditional family values. Notwithstanding, this plurality of views reflects foundational controversies over family values and approaches to pediatric decision making. And it seems like we are stuck with a debate in which either you favor the best interests of the child (from a liberal perspective) or reject that parents are mere trustees of their children (from a traditional perspective). We have shown that we can make progress beyond this intractable disagreement embedded in conflicting worldviews. Systemic thinking has revealed that the stability of the family itself depends on involving all family members. It is in the interest of the family to resist the initial aversive reaction to delivering bad news.

## Recommendations

It is unlikely that the child and the family will be prevented from the salient and burdening aspects of a poor, deteriorated, or terminal prognosis. Yet, sometimes, pediatric oncology providers dislike to refuse parental wishes for fear of further destabilizing the family unit. The perception of making matters worse may be understandable, but we should not be content with staying away from the situation. As Erica Brown puts it: “There is no way of dressing up the harsh facts of a child’s diagnosis of life-limiting illness (…). However, with skilled support, family members can develop strategies that help them (…) to function as an integrated team, supporting one another.” (Brown and Warr 2007, p. 85).

We argued that the stability of the family argument cannot serve as a general justification for withholding information from children. Thus, pediatric oncology providers have to take additional considerations into account when evaluating disclosure, such as a child’s maturation, a child’s developing autonomy, a child’s best interest, a child’s awareness of its reality, family dynamics, a family’s communication and interaction patterns, a family’s cultural background, the type of treatment decision in question, siblings’ needs, or familial long-term coping. This more comprehensive outlook can break down the narrow perception that only the prevention of stress from delivering bad news is what matters. Pediatric oncology providers may feel liberated to find out that there are more options to deal with the family’s need for stability.

To counter parental requests for non-disclosure, a Swiss study suggested pediatric oncology providers should proactively address diagnostic disclosure: “physicians [should] employ a proactive stance in ensuring that children with cancer are appropriately informed about their diagnosis.” (Badarau et al. 2015, p. 2177). Pediatric oncology providers have an obligation to answer honestly possible questions of the child—lying is not an option (Cole and Kodish 2013). Therefore, in cases of children who sense their reality and who want to know, revelation seems inevitable. The proactive stance, however, should be attentive to a family’s idiosyncratic interaction and communication patterns from diagnosis onwards which, ultimately, facilitates communication in case of a deteriorated prognosis. Here, systemic approaches help to broaden the perspective on the family (e.g. family dynamics that extend beyond dyadic interactions, long-term functioning of a family) and to identify further important considerations (e.g. siblings’ coping, effects of disclosure).

For the individual child’s perspective on communication during cancer, a recent review spanning 101 articles from across 25 countries concluded: “patients gain a sense of respect, safety, and control when they feel clinicians address their information and developmental needs. However, communication that is perceived to be parent-centered can be disempowering. Promoting child agency and partnership may improve care and outcomes for children with cancer.” (Lin et al. 2019, p. 701) This conclusion highlights the benefits that prognostic disclosure can (and frequently does) have for the child. Pediatric oncology providers should therefore emphasize the respective evidence when discussing a child’s involvement with the parents. Parents, most likely, will aim to avoid disempowering experiences on the part of their child and might even be thankful for being made aware of the benefits of open disclosure. Complete, balanced, and accurate information is crucial for parental decision-making.

Attitudes towards information disclosure are strongly determined by a family’s cultural background (Rost et al. 2019; Mitchison et al. 2012; Shahidi 2010; Mystakidou et al. 2005; Wiener et al. 2013). Moreover, it was shown that ethnic minorities’ are less likely to recognize poor prognosis and unlikely cure (Mack et al. 2020). Emphasizing that “illness behavior is a normative experience governed by cultural rules”, Kleinman and colleagues point to “’approved’ ways of being ill”, which are learned by individuals, including how illness is coped with (Kleinman et al. 1978, p. 141). Our analysis is particularly relevant for culturally diverse countries, such as Switzerland (Swiss Federal Statistical Office 2018), in which pediatric oncology providers are more likely to treat children with a different cultural background. Pediatric oncology providers have to consider what cultural norms structure family dynamics.

Moreover, what makes parents request non-disclosure is maybe a fixed and exaggerated interpretation of their role of a protector of the child. They see themselves only as safeguards against potentially distressing information. However, the stability of the family is not secured when parents exclusively fulfill a protector role. Systemic thinking suggests that parents need to be more flexible with what roles they can fulfill. They could act as a mediator who filters information, individually tailors information to the individual child (whom they know best), and takes into account the entire family unit’s need to adapt to the situation at hand. Pediatric oncology providers could sensitize parents for this broadened—family unit—perspective.

Lastly, pediatric oncology providers appreciate the importance of moral and practical competence (Weiner et al. 2020). For example, you need to understand the complexity of moral challenges, identify the essence of the moral challenge, know what information to convey to the child and their family and be “brave and confident enough to speak up and support others to also express their opinions on moral grounds” (Weiner et al. 2020, p. 9). When facing moral challenges, pediatric oncology providers should not hesitate to involve ethics consultation to clarify the moral question at hand. Besides, other occupational groups (e.g. psycho-oncologists, social workers) should be involved in intra-team decision-making to better understand the family situation from a systemic perspective.

Summing up, recommendations based on our analysis of the stability of the family argument are (1) taking additional considerations into account when evaluating prognostic disclosure to the child, (2) proactively addressing prognostic disclosure and being attentive to a family’s interaction and communication patterns, (3) highlighting the possible beneficiary impact of prognostic disclosure, (4) ensuring cultural safety by recognizing a family’s beliefs and values, (5) broadening the parental perspective beyond the child and their role as protectors of the child, (6) involving ethical consultation and other occupational groups. While these steps do not assure arriving at a consensus on prognostic disclosure, they provide tangible decision-making aids for preventing and dealing with parents’ request for non-disclosure.

## Conclusion

The stability of the family argument is based on a highly problematic estimation of potential risks. Hence, the general worry of instability is unwarranted. The family argument’s conclusion is false because non-disclosure of prognostic does not by itself contribute to the stability of the family. It is therefore of limited value for DM in the pediatric setting.

Blanket use of a presumed destabilization can even create further barriers to DM in the pediatric setting. Pediatric oncology providers presumably faced decisional uncertainty when being confronted with parental wishes not to disclose information to the child. However, they opted to not disclose prognostic information to the child. One possible explanation is motivated reasoning (Kunda 1990). Acknowledging the role motivated reasoning might have played can be substantiated by studies reporting that physicians do not consistently articulate prognosis to families (Mack et al. 2007; Rosenberg et al. 2014; Durall et al. 2012). Under decisional uncertainty, which might even evoke moral distress, pediatric oncology providers might have arrived at conclusions they wanted to arrive at, namely the conviction that non-disclosure is beneficial for the family. Besides, parents have legal and ethical decisional authority, which also increases the likelihood of—legally accountable—physicians following parental wishes. The stability of the family argument may be useful on the part of health professionals to protect themselves from moral distress, legal complications, and decisional ambiguity. But if we want to preserve the stability of the family in adverse situations we should move away from the assumption that a child’s wellbeing means to shield them from bad news. We need to develop strategies that manage family dynamics through open dialogue.

## Data Availability

Not applicable.
